# Editorial: Metabolomics in personalized cancer medicine

**DOI:** 10.3389/fmolb.2025.1593877

**Published:** 2025-04-08

**Authors:** Joana Pinto, Jacopo Troisi

**Affiliations:** ^1^ Associate Laboratory i4HB-Institute for Health and Bioeconomy, University of Porto, Porto, Portugal; ^2^ UCIBIO-Applied Molecular Biosciences Unit, Department of Biological Sciences, Laboratory of Toxicology, Faculty of Pharmacy, University of Porto, Porto, Portugal; ^3^ Department of Medicine, Surgery and Dentistry, “Scuola Medica Salernitana”, University of Salerno, Baronissi, Campania, Italy; ^4^ Theoreo srl, Montecorvino Pugliano, Campania, Italy; ^5^ European Institute of Metabolomics (EIM) Foundation, Baronissi, Campania, Italy

**Keywords:** metabolomics, personalized medicine, evidence based medecine, biomarker, personalized oncology, cancer

Evidence-Based Medicine (EBM) is widely recognized as one of the greatest achievements of modern medical science (Straus et al., 2019). It has led to significant public health outcomes since the replacement of the paternalistic approach to medical practice in the mid-19th century. To take just one example, in the last 150 years, average life expectancy has more than doubled, while infant mortality has fallen from over 40% to less than 0.3% (Zijdeman and Ribeiro de Silva, 2014). Despite the significant statistical improvement, there is a broad consensus within the scientific community that EBM must evolve toward Personalized Medicine as an emerging clinical paradigm. EBM focuses on the disease itself rather than on the individual, grouping participants in clinical trials according to the uniformity of their pathological conditions. In contrast, Personalized Medicine focuses on the uniqueness of each person, their personal history and their ability to cope with disease (Passaro et al., 2024). In this paradigm shift, understanding human individuality requires an inherently holistic perspective, which can only be achieved through an “omics” approach. Personalized Medicine aims to provide prevention and treatment strategies tailored to each patient’s unique genetic and phenotypic profile. To date, genomic profiling has played a pivotal role in guiding therapeutic decisions to minimize treatment failure, relapse, and adverse drug effects (Sadee et al., 2023). However, it is increasingly evident that integrating multiple “omics” technologies—ranging from transcriptomics to proteomics and metabolomics—can provide a more comprehensive understanding of the biological processes underlying cancer, allowing the identification of novel diagnostic and therapeutic targets.

Among these multi-omics approaches, metabolomics offers unique advantages through the precise quantification of biochemical phenotypes across multiple biological scales–from intracellular metabolic alterations within tumor tissue to systemic metabolic response in patients undergoing therapeutic intervention (Jacob et al., 2019). Malignant cells profoundly rewire their metabolic machinery to support proliferation, growth, and invasion, as well as to develop resistance to treatment. Metabolic profiling enables the detection of these metabolic perturbations at the small molecule level, facilitating the identification of biochemical signatures that can serve as predictive biomarkers for therapeutic response, susceptibility to adverse events, and the likelihood of disease recurrence.

Rapid advances in analytical techniques (e.g., mass spectrometry and nuclear magnetic resonance spectroscopy) and data processing (boosted by artificial intelligence and big data analytics) are opening up new prospects for truly personalized therapeutic protocols. In this context, the integration of metabolomic data with information derived from genomics, transcriptomics, and proteomics further enhances diagnostic accuracy and the ability to define targeted, dynamic strategies that can adapt to the evolution of each tumor.

This Research Topic on “*Metabolomics in Personalized Cancer Medicine*” presents state-of-the-art research advancements, innovative methodological approaches, and ongoing challenges in applying metabolomics to precision oncology. This Research Topic comprises five research articles covering different areas of oncology, including diagnosis, therapeutic interventions, and treatment-related complications. Collectively, these studies demonstrate how metabolomics can elucidate cancer-specific metabolic signatures in different malignancies, thereby providing a fundamental framework for Personalized Medicine approaches in oncology.

The study by Feng et al. used a Mendelian randomization (MR) approach to evaluate causal associations between blood plasma metabolites and prostate cancer risk. Analysis of 1,091 plasma metabolites and 309 ratios revealed 152 significant causal associations with prostate cancer risk, of which 20 remained consistent across two independent datasets. In particular, three metabolic ratios - arginine/glutamate, phosphate/uridine and glycerol/mannitol/sorbitol - showed consistent inverse associations with prostate cancer risk, suggesting their potential utility as biomarkers. This study also showed a significant association between glutathione metabolism, driven by N-acetyl-L-glutamine, and prostate cancer development. These findings may have implications for the development of new prevention and treatment strategies for prostate cancer.

The study by Jia et al. also used MR to identify blood metabolites causally associated with an increased risk of esophageal cancer. Out of 486 blood metabolites, a significant association was found between esophageal cancer and three metabolites, specifically laurate (12:0), 1-linoleoyl-sn-glycero-3-phosphoethanolamine, and pyroglutamine. These associations were then validated by meta-analysis using datasets from several populations. The study suggested that esophageal cancer is linked to three altered metabolic pathways involving fatty acid biosynthesis and beta-oxidation, with laurate (12:0) playing a critical role in all pathways. These findings advance esophageal cancer research by suggesting new avenues for prevention and early diagnosis.

The study by Yagin et al. focused on the discovery of breast cancer biomarkers by integrating targeted metabolomic data with machine learning and explainable artificial intelligence tools to build interpretable predictive models. Leucine, isoleucine, L-alloisoleucine, norleucine and homoserine were the most promising biomarkers for breast cancer detection, with a combined high performance (89.5% precision, 88.4% recall and 83.7% specificity). Significant correlation coefficients were also found between these metabolites (e.g., isoleucine/L-alloisoleucine, leucine/norleucine). This research provides clinically interpretable metabolic biomarkers that could improve early breast cancer detection and treatment planning.

Hassanein et al. focused on triple-negative breast cancer, investigating how chemotherapeutic agents—doxorubicin, 5-fluorouracil, and their combination—alter the tumor and plasma metabolomes using a xenograft mouse model. The researchers identified distinct metabolic profiles in tumor and plasma for each treatment, with the combination therapy causing the most extensive disruptions in purine, pyrimidine, beta-alanine and sphingolipid metabolism. The research identified candidate biomarkers for chemotherapy response (L-fucose and beta-alanine), providing insights into how triple-negative breast cancer tumors metabolically adapt to chemotherapy, contributing to more targeted treatment strategies and Personalized Medicine approaches.

Finally, the study by Kirkwood-Donelson et al. used untargeted metabolomics to investigate erastin-induced ferroptosis in ovarian cancer cells. The researchers identified several potential biomarkers of ferroptosis, including increased ophthalmate formation and decreased levels of glutathione, taurine, NAD^+^/NADP^+^, carnitines, and glutamine/glutamate. These metabolic signatures may help predict the response to ferroptosis-inducing treatments, potentially enabling more personalized and effective approaches for ovarian cancer patients who often develop resistance to conventional therapies. By identifying these metabolic biomarkers, clinicians may be able to better identify patients who would benefit from ferroptosis-based treatments.

Together, these studies highlight the critical importance of identifying metabolite-based biomarkers and altered metabolic pathways across multiple cancers. By using advanced data analytics, and translating findings into actionable biomarkers, these studies provide a coherent path towards more precise, effective and personalized cancer care. However, the translation of these research findings into clinical practice will require coordinated efforts extending beyond the laboratory. The refinement of analytical protocols, the standardization of data-sharing platforms, and the promotion of interdisciplinary partnerships should be pursued jointly by clinicians, scientists, industry partners, and policymakers. The integration of metabolomics with other “omics” technologies remains a critical next step for comprehensive cancer profiling. These initiatives will help to accelerate the development of robust metabolomics-based tools that can be integrated into routine medical workflows.

In addition, continued investment in education and training is essential to enable healthcare providers to take full advantage of Personalized Medicine. By creating a healthcare ecosystem in which therapies are tailored to each patient’s unique biological profile, treatment outcomes can be improved, adverse effects mitigated, and resource allocation optimized. With each scientific breakthrough, Personalized Medicine moves from a theoretical concept to a standard of care, revolutionizing how we understand and treat cancer.
